# Recognition of interferon-inducible sites, promoters, and enhancers

**DOI:** 10.1186/1471-2105-8-56

**Published:** 2007-02-19

**Authors:** Elena A Ananko, Yury V Kondrakhin, Tatiana I Merkulova, Nikolay A Kolchanov

**Affiliations:** 1 Institute of Cytology and Genetics SB RAS, Lavrentiev av., 10, 630090 Novosibirsk, Russia; 2 Institute of Systems Biology, Novosibirsk, Russia; 3 Design Technological Institute of Digital Techniques SB RAS, Novosibirsk, Russia

## Abstract

**Background:**

Computational analysis of gene regulatory regions is important for prediction of functions of many uncharacterized genes. With this in mind, search of the target genes for interferon (IFN) induction appears of interest. IFNs are multi-functional cytokines. Their effects are immunomodulatory, antiviral, antibacterial, and antitumor. The interaction of the IFNs with their cell surface receptors produces an activation of several transcription factors. Four regulatory factors, ISGF3, STAT1, IRF1, and NF-κB, are essential for the function of the IFN system. The aim of this work is the development of computational approaches for the recognition of DNA binding sites for these factors and computer programs for the prediction of the IFN-inducible regions.

**Results:**

We developed computational approaches to the recognition of the binding sites for ISGF3, STAT1, IRF1, and NF-κB. Analysis of the distribution of these binding sites demonstrated that the regions -500 upstream of the transcription start site in IFN-inducible genes are enriched in putative binding sites for these transcription factors. Based on selected combinations of the sites whose frequencies were significantly higher than in the other functional gene groups, we developed methods for the prediction of the IFN-inducible promoters and enhancers. We analyzed 1004 sequences of the IFN-inducible genes compiled using microarray data analyses and also about 10,000 human gene sequences from the EPD and RefSeq databases; 74 of 1,664 human genes annotated in EPD were significantly IFN-inducible.

**Conclusion:**

Analyses of several control datasets demonstrated that the developed methods have a high accuracy of prediction of the IFN-inducible genes. Application of these methods to several datasets suggested that the number of the IFN-inducible genes is approximately 1500–2000 in the human genome.

## Background

Computational analysis of the genomes in combination with large-scale expression studies becomes increasingly important for detailed functional annotation of the eukaryotic and prokaryotic genomes. Analysis of regulatory regions of protein-coding genes is an important aspect of functional annotation. The most frequently used approach to the study of the regulatory regions of genes relies on search of the so-called transcriptional regulatory modules. These modules are the gene regions enriched in the binding sites (BSs) for a set of particular transcription factors (TFs). The underlying idea is that a specific transcription of a particular group of genes is regulated by multiple interactions of a set of TFs. At present, several methods for the identification of the transcriptional regulatory modules are available. These include, for example, ModuleSearcher and ModuleScanner [1], CONFAC [2], MSCAN [3], Composite Module Analyst [4].

A biomedically important aspect of the functional annotation of the human genome is the identification of the interferon (IFN)-controlled genes. The IFNs are cytokines that possess diverse biological properties. IFNs of the type I, IFNs-α and IFN-β activate cells of the immune system and modulate cell differentiation [5]. The IFN of the type II, IFN-γ, is important for the development of antibacterial and antiparasitic immune responses [6]. It has been also shown that it is involved in the development of some autoimmune diseases [7]. The interaction of the IFNs with cell surface receptors activates the JAK-STAT signal transduction pathway resulting in the activation of the TFs ISGF3 (the type I IFNs) and STAT1 (the type II IFN) [5,8,9]. Some members of the IRF family of TFs [10] are also critical for functioning of the IFN system [11]. These IRFs regulate the cell cycle; they also affect antigen presentation and production of nitric oxide [12]. Interactions of the ISGF3, STAT1, and IRFs with their BSs in the regulatory regions of the IFN-stimulated genes (ISGs) enhance their transcription.

Despite the long-standing studies of the IFN system, the intricate mechanisms of its function and the contribution of individual ISGs to the development of the immune response remain unclear in many aspects. Moreover, by far not all the ISGs have been identified as yet. In particular, support for this comes from the discrepancy between the reported estimates of the ISG number in the human genome, more than 1000 [13,14], and the dozens well studied.

The goal of this study was to reveal the transcriptional regulatory modules that are most specific to the ISGs of three types: the arbitrary IFN-inducible genes (stimulated by any IFN), the genes induced by the type I IFNs, and the genes stimulated by the type II IFNs. We chose a way different from the currently used approaches, which are based on universal descriptors applicable to diverse gene families. MSCAN [3] and Composite Module Analyst [4] are good examples of bioinformatics tools that use such methods. We abandoned universality for a more detailed analysis of IFN-stimulated genes through identification of their most specific BS patterns. Instead of the sophisticated methods for a universal description of the transcriptional regulatory modules, we preferred relatively simple recognition methods adapted for analysis of specific classes of IFN-inducible modules. The improved detection of the potential ISGs by the computer-assisted approaches might provide a better understanding of their underlying mechanisms and side effects of the popular IFN therapy.

## Results and discussion

### Distribution of the IRF1, ISGF3, STAT1, and NF-κB binding sites in the ISG sequences

**Figure 1:**
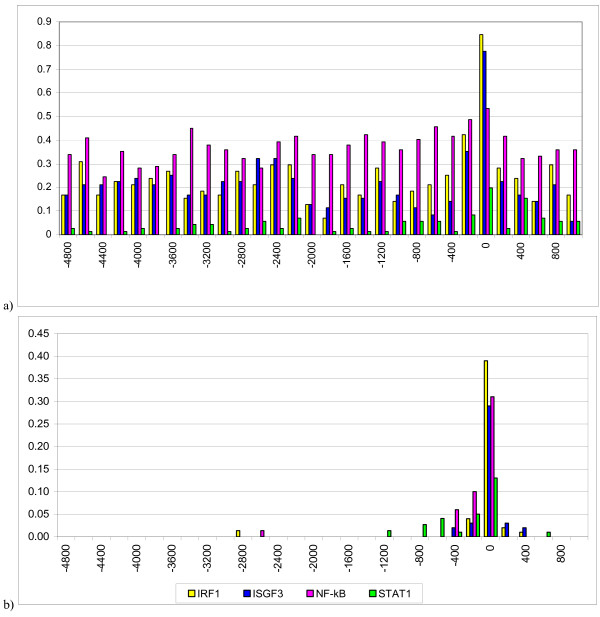
**The occurrence frequencies of the IRF1, ISGF3, STAT1, and NF-κB binding sites in the 5'-flanking regions from the training-ISG set with respect to the TSS**. The TSS is designated as 0 along the X-axis; Y-axis, the occurrence frequency of a BS within 200 bp region normalized for gene number in the sample; a) putative binding sites; b) true experimentally confirmed sites.

We developed methods for the recognition of the 20 BSs including BSs for TF IRF1, ISGF3, STAT1, and NF-κB, the key regulators of the IFN system. For each BS type, we included all available experimentally verified sites. Each sample contained at least 30 BSs. To develop the methods, we chose only those site types that are known to have the strongest effects at the transcription level of ISGs. Using the developed methods (described in the Methods section), the localization patterns of the putative BSs in the regions spanning from -5000 to +2000 bp with respect to the TSS in the training sample of the ISGs (training-ISG set) were analyzed (Fig. 1a). It is generally accepted that the promoter regions are enriched in BSs for various TFs. For IRF1, ISGF3, and STAT1, the density of the putative BSs in the region from -200 to +1 bp with respect to the TSS is twofold higher than in the rest of the region. In this region, the putative BSs for IRF1 and ISGF3 were present in the majority of genes (85% and 77%, respectively) and STAT1 BSs were detected in 20% of the genes. By contrast, no considerable increase in the density was observed in the region near the TSS for the putative NF-κB BSs. In recognition of the putative BSs here and further, in addition to the common threshold, we used the additional threshold values a_2_* needed for statistical simulation (see Methods). They were adapted in a way to maximally reduce the number of false positives by omitting not more than half of the actual sites of the training samples. Stating it otherwise, reliability of the predicted sites was provided by reasonable omission of actual, but weak, sites.

It should be noted that the experimentally confirmed BSs for IRF1, ISGF3, and STAT1 were identified in 39%, 29%, and 13% of these genes, respectively (Fig. 1b). This disagreement between the number of the putative and experimentally confirmed BSs suggested that a substantial fraction of these genes have been poorly studied, thus many of their BSs remain unknown. This applies not only to the ISGs, novel BSs are increasingly revealed in the regulatory regions of many genes. A number of undetected BSs may be especially large for regulatory regions located in great distances from the TSSs. To confirm this, we refer to the results of ChIP-chip data [15] analysis. It has been found that only 15% of the experimentally detected BSs for TF MYC are at a distance of <200 bp from the TSS, 33% at <1000 bp, and 49% are by more than 10000 bp away from the TSS. However, the BSs for MYC stored in TRANSFAC are differently located relative to the TSS, namely 56% of the BSs are at a distance of <200 bp from the TSS, 67% are at <1000 bp, and none is further away than 10000 bp from it. A similar distance distribution was observed for the MYC BSs stored in TRRD. Thus, in terms of the experimentally verified MYC BSs from the TRANSFAC and TRRD databases, the site location near the TSS prevails. By contrast, in terms of the ChIP-chip experiments, there is no such prevalence. The reason for the discrepancy is not the poor annotation of the BSs in the TRANSFAC or TRRD databases. The reason is that many genes are still insufficiently studied. As a rule, until recently, researchers focused their attention on the promoter regions, and the regions far beyond TSSs that contain the functional sites were often left unnoticed.

In general, presence of unknown remote regulatory regions might cause substantial problems for computational analyses. However, our study of the ISG promoters demonstrated that the vast majority of BSs are located near TSSs. To delineate the structural features of ISGs, the regulatory regions of 3 functional groups of genes were compared. The gene regions spanning from -500 to +500 bp with respect to the TSSs were examined; 1664 random human promoters from the EPD (control-EPD set) were used as a control. There was a severalfold density excess of the putative BSs for the IRF1, ISGF3, and STAT1 in the [-400; -1] region of ISGs (see Additional file 1: Distribution of IRF1, ISGF3, STAT1, and NF-κB binding sites in different gene groups), whereas the density excess was less pronounced in some other regions (data not shown). For this reason, we further analyzed the [-500; +300] region that was found to be important in determination of the specificity of the ISG regulatory regions. Omission of the sites included in the training samples for site recognition did not affect the distribution feature (Table 1). Differences between the training-ISG set and the other gene groups are shown in Table 1.

**Table 1:** Relative occurrence frequencies of the BSs in the promoter regions of genes grouped according to functional activity

	Relative occurrence frequency of a site in a sample					
			
BS (position with respect to the TSS)	training-ISG set	training-ISG set excl.*	control-Gluco set	control-LipM set	control-EPD set	ISGs/EPD ratio
IRF1 (-200 to -1)	0.77	0.70	0.21	0.18	0.21	3.67
ISGF3 (-100 to -1)	0.28	0.19	0.00	0.00	0.05	5.60
ISGF3 (-200 to -1)	1.00	0.85	0.08	0.17	0.10	10.00
STAT1 (-200 to +1)	0.19	0.15	0.05	0.00	0.08	2.38
NF-κB (-100 to +100)	0.93	0.80	0.46	0.46	0.12	2.02

*BSs used for drawing training samples for site recognition were omitted

Correct regulation of the gene expression is provided not only by the presence of BSs of particular types, it also requires specific localization of BSs with respect to TSSs and each other. In the detection of the characteristic features of the BS localization in ISGs, not only the key regulators, but also their possible interactions with the other regulators must be taken into account. In addition to the distribution of the main regulators (see Additional file 1: Distribution of IRF1, ISGF3, STAT1, and NF-κB binding sites in different gene groups), we checked the distribution of the putative BSs for another 14 TFs, namely, AP1, C/EBP, E2F, GATA1, GR, HNF1, HNF3, HNF4, MyoD, NF-Y, OCT1, SF1, Sp1, and TATA-box. Only a few BSs (AP1, GATA1, OCT1, Sp1, and TATA-box) showed the higher density specific to the ISGs in the region of interest. Next, we turned to analysis of the frequencies of the sites for the main regulators and these TFs. About 4 hundreds of various site combinations were examined. Emphasis was on the combinations of the putative BSs of the key regulators and also their combinations with the sites of the other types. Of these, 158 combinations whose occurrence frequencies in the training-ISG set were significantly different from these in the control sets were selected. Most BS pairs were composed of combinations involving BSs of the key regulators, including their tandem repeats. Taking advantage of individual sites, their combinations and information about the type and level of induction of each gene of the training-ISG set, we developed 3 methods for the recognition of the IFN-inducible regions in DNA. In order to do this, we compiled two subsamples from the training-ISG set; the genes mainly induced by the IFNstypeI (training-ISG subset 1) were assigned to the first subsample and those by the IFNγ to the second subsample (training-ISG subset 2). The recognition methods for the IFN-inducible regions were as follows:

i. method 0, any IFN-inducible region (induction by IFN of any type);

ii. method 1, promoter or regulatory region inducible by type I IFNs (IFNα, IFNβ);

iii. method 2, promoter or regulatory region of the type II IFN (IFNγ) -inducible genes.

Each method was based on the patterns obtained by comparison of the respective training-ISG set and subsets 1,2 with the control-EPD set. The patterns selected for each method are given in Tables 2, 3, and 4, respectively. The resulting patterns were the individual sites with defined disposition relative to TSS or a combination of two sites. Individual sites were identified using the asymptotical statistical test for comparison of two binomial variables. Site pairs were selected using the standard χ^2 ^test based on the 2 × 2-contingency tables (for details, see the Methods section 'Building of recognition methods for the interferon-inducible promoters and enhancers').

**Table 2:** Patterns for method 0, recognition of any IFN-inducible DNA region (stimulation by any IFN)

Site 1	Site 2	W = F1/F2 (weight for the pattern)
strongISGF3 (b)* from -350 to +350**	-	8.57
strong ISGF3 (+) from -350 to +350	-	10.48
ISGF3 (b) from -200 to +50	-	9.41
ISGF3 (+) from -200 to +50	ISGF3 (+) from -500 to +1	9.41
strong ISGF3 (+) from +130 to +350	ISGF3 (+) from +130 to +350	3.14
strong STAT1 (-) from -350 to +350	-	9.36
STAT1 (-) from -350 to +350	-	3.66
STAT1 (-) from -200 to +50	STAT1 (+) from -350 to +350	3.76
STAT1 (-) from +130 to +350	-	3.22
strong IRF1 (b) from -350 to +350	-	5.0
IRF1 (-) from -650 to +350	-	5.18
IRF1 (-) from -200 to +50	IRF1 (-) from +130 to +350	3.50
AP1 (+) from -500 to -300	-	1.20
AP1 (b) from -300 to +1	-	1.13
NF-Y (-) from -150 to +50	-	1.28
OCT1 (+) from -300 to +50	-	1.31
NF-κB (-) from -300 to +1	NF-κB (+) from -300 to +1	5.16
ISGF3 (b) from -200 to +50	STAT1 (+) from -350 to +350	3.64
STAT1 (b) from +130 to +350	TATA (+) from -100 to +70	3.50
STAT1 (b) from -300 to +1	TATA (+) from -100 to +70	13.0
STAT1 (-) from +130 to +350	TATA (+) from -100 to +70	3.0
AP1 (b) from -300 to +1	TATA (+) from -100 to +70	2.0
STAT1 (+) from -350 to +350	IRF1 (b) from -300 to +1	3.74
IRF1 (+) from -350 to +350	ISGF3 (b) from -500 to +1	5.1
strong ISGF3 (b) from -600 to +350	STAT1 (b) from -650 to +350	10.8
ISGF3 (b) from -200 to +50	STAT1 (b) from -650 to +350	5.8
strong IRF1 (b) from -650 to +350	STAT1 (b) from -650 to +350	10.8
strong IRF1 (b) from -650 to +350	strong ISGF3 (b) from -600 to +350	5.8
ISGF3 (b) from -200 to +50	strong IRF1 (b) from -650 to +350	3.8
strong ISGF3 (b) from -600 to +350	IRF1 (b) from -500 to +1	4.8
ISGF3 (b) from -200 to +50	IRF1 (b) from -500 to +1	4.8
NF-κB (b) from -500 to +1	strong IRF1 (b) from -650 to +350	8.8
NF-κB (b) from -500 to +1	strong IRF1 (b) from -500 to +1	3.8
NF-κB (b) from -500 to +1	AP1 (b) from -500 to +1	1.8
IRF1 (b) from -200 to +50	NF-κB (b) from -500 to +1	2.0
AP1 (b) from -500 to +1	strong IRF1 (b) from -650 to +350	3.8
AP1 (b) from -500 to +1	IRF1 (b) from -500 to +1	1.8
ISGF3 (b) from -200 to +50	OCT1 (b) from -500 to +1	1.4
STAT1 (+) from -300 to +1	OCT1 (b) from -500 to +1	1.3

*b (both) - any direction of a site; + forward DNA strand; - reverse DNA strand;

** Location of a site with respect to the TSS

**Table 3:** Patterns for method 1, recognition of DNA regions induced by type I IFNs (IFNα, IFNβ)

Site 1	Site 2	W = F1/F2 (weight for the pattern)
strongISGF3 (b)* from -350 to +350**	-	3.25
ISGF3 (b) from -200 to +50	-	3.89
ISGF3 (b) from +130 to +350	-	1.83
STAT1 (+) from -350 to +350	-	2.29
STAT1 (b) from -300 to +1	-	1.68
STAT1 (-) from +130 to +350	-	1.67
strong IRF1 (b) from -350 to +350	-	2.76
strong IRF1 (+) from -350 to +350	-	2.0
IRF1 (+) from +130 to +350	-	2.16
strong NF-κB (-) from -300 to +1	NF-κB (-) from -300 to +1	1.8
IRF1 (b) from -350 to +350	ISGF3 (b) from -200 to +50	7.64
IRF1 (b) from -500 to -300	ISGF3 (+) from -200 to +50	4.25
IRF1 (b) from -300 to +1	STAT1 (b) from -350 to +350	9.4
ISGF3 (b) from -200 to +50	AP1 (b) from -500 to +1	3.74
IRF1 (b) from -200 to +50	GATA1 (b) from -600 to +1	3.45
IRF1 (+) from -350 to +350	GATA1 (b) from +45 to +350	2.1
ISGF3 (b) from -200 to +50	OCT1 (b) from -500 to +1	2.3
ISGF3 (b) from -200 to +50	GATA1 (b) from -600 to +1	2.35
ISGF3 (b) from -200 to +50	AP1 (b) from -500 to +1	5.31
ISGF3 (b) from -200 to +50	OCT1 (b) from -500 to +1	1.92
STAT1 (b) from -350 to +350	ISGF3 (b) from -200 to +50	4.53
STAT1 (b) from -500 to +1	GATA1 (b) from +45 to +350	1.92
STAT1 (+) from -350 to +350	OCT1 (b) from -500 to +1	2.66

*b (both) - any direction of a site; + forward DNA strand; - reverse DNA strand;

** Location of a site with respect to the TSS

**Table 4:** Patterns for method 2, recognition of DNA regions stimulated by the type II IFN (IFNγ)

Site 1	Site 2	W = F1/F2 (weight for the pattern)
strong STAT1 (-) from -350 to +350	STAT1 (-) from -350 to +350	20.00
STAT1 (-) from -350 to +350	-	9.36
STAT1 (-) from +130 to +350	-	4.22
strong IRF1 (b) from -350 to +350	IRF1 (b) from -350 to +350	10.0
IRF1 (-) from -350 to +350	AP1 (b) from -500 to +1	5.4
IRF1 (b) from -650 to +350	GATA1 (b) from -600 to +1	1.17
IRF1 (b) from -200 to +50	NF-κB (b) from -500 to +1	6.0
IRF1 (+) from -500 to +1	OCT1 (b) from -500 to +1	2.0
IRF1 (+) from -350 to +350	TATA (+) from -100 to +70	5.2
STAT1 (-) from -350 to +350	strong IRF1 (b) from -650 to +350	10.0
strong STAT1 (-) from -350 to +350	STAT1 (-) from -350 to +350	20.00
STAT1 (-) from -350 to +350	-	9.36
STAT1 (-) from +130 to +350	-	4.22
strong IRF1 (b) from -350 to +350	IRF1 (b) from -350 to +350	10.00
STAT1 (b) from -500 to +1	strong IRF1 (b) from -650 to +350	6.1
STAT1 (b) from -500 to +1	ISGF3 (b) from -500 to +1	2.1
STAT1 (+) from -350 to +350	AP1 (b) from -500 to +1	3.8
STAT1 (+) from -300 to +1	OCT1 (b) from -500 to +1	3.3
STAT1 (b) from -350 to +350	NF-κB (b) from -500 to +1	4.1
STAT1 (-) from -130 to +350	TATA (+) from -100 to +70	4.0
STAT1 (b) from -300 to +1	TATA (+) from -100 to +70	8.0
NF-κB (b) from -500 to +1	AP1 (b) from -500 to +1	5.4

*b (both) - any direction of a site; + forward DNA strand; - reverse DNA strand;

** Location of a site with respect to the TSS

Dashes in the second columns of Tables 2, 3, and 4 indicate the individual sites. The weight of each pattern, W = F1/F2 (F1 is the occurrence frequency in the training-ISG set, F2 that in the EPD-control set, see Methods) is next to it. The weight indicates how many times more frequently the pattern occurs in the training-ISG set compared to the control-EPD set. Tables 2, 3, and 4 contain all the selected patterns whose occurrence frequencies in the training-ISG set significantly exceed those in the control-EPD set.

To check how the methods work, information from 16 articles on the ISGs identified by microarrays (Additional file 2: List of references for drawing the ISGs samples from microarray data) was assessed. Based on this information, we compiled a sample of the promoter regions of 1005 ISGs (the microarray-ISG set). However, the information about the type and extent of IFN induction for some of the genes was incomplete. For this reason, two subsamples, microarray-ISG subset 1 and microarray-ISG subset 2, were derived from microarray-ISG set. The microarray-ISG subset 1 contained only gene sequences whose induction by the type I IFNs (IFNα, IFNβ) was more than twofold during the first 12 hours of IFN stimulation. Thus, most genes whose induction by IFNs might have been caused indirectly and also the "weak" ISGs were ignored. This made the microarray-ISG subset 1 (668 genes) contain less falsely included genes than the microarray-ISG set. The microarray-ISG subset 2 was composed of the sequences of the genes induced by the type II IFN; the restrictions imposed on their inclusion for the microarray-ISG subset 2 were the same as for the microarray-ISG subset 1. Since there were much less data for the fold induction by the IFNγ than for the type I IFNs, microarray-ISG subset 2 ultimately contained 97 genes *versus *the 668 genes in the microarray-ISG subset 1.

What if the microarray-ISG set contained some "weak" ISGs and/or falsely included genes? This may be the case because only 23.3% of the genes were recognized (Table 5). By contrast, in the microarray-ISG subset 1 and microarray-ISG subset 2, recognition was much better and comparable to that in the training-ISG-set for both the short and long sequences. This result suggested that the microarray-ISG subset 1 and the microarray-ISG subset 2 were quite homogenous and representative.

**Table 5:** Recognition of the IFN-inducible DNA regions in the microarray-derived genes

Sample	-5000 to +2000*	-1000 to +1000
Microarray-ISG set	23% (31%)**	17.5% (27%)
Microarray-ISG subset 1	49% (54%)	24% (26%)
Microarray-ISG subset 2	37% (37%)	25% (26%)

* Sequence size in a sample with respect to TSS

**The percentage for the ISG-training set is in parentheses

Microarray-ISG subset 1, IFN type I-inducible ISGs

Microarray-ISG subset 2, IFN type II-inducible ISGs

At restricted sequence length, the recognition was poorer because the IFN-inducible enhancers could be far away from TSSs. ISGs containing enhancers at the distance of ~1000 bp upstream of TSSs were not recognizable after sequences were truncated.

**Figure 2:**
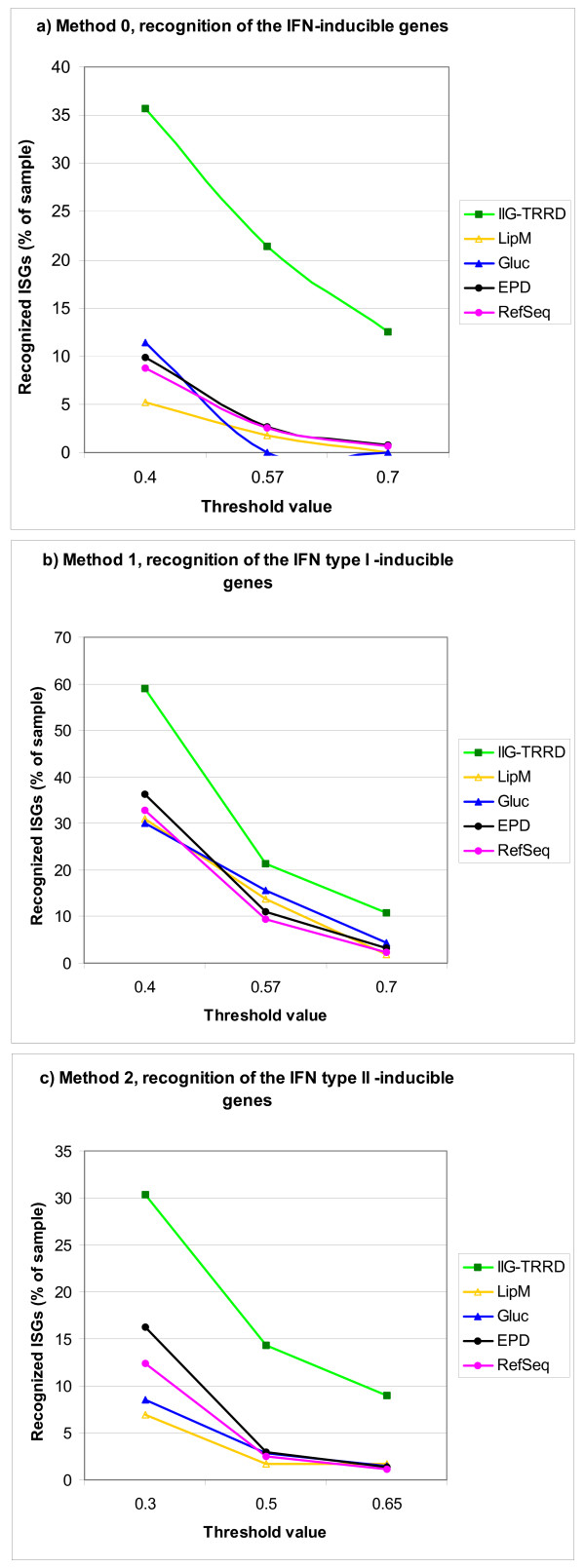
**Dependence of recognition accuracy on the function threshold**. a) method 0; b) method 1; c) method 2.

Using the developed methods at various threshold levels, DNA sequences from -1000 to +1000 bp with respect to the TSS from gene groups were analyzed. The dependence of recognition on the function threshold value is graphically represented in Fig. 2. Method 2 outperformed the other two: it recognized the regions responsive to any IFN where, even at the maximum cut-off (0.7), about 1% of the genes were recognized in the EPD and RefSeq samples, but recognition remained high, at 12.5%, in the training-ISG set (Fig. 2a). Method 2 (recognition of the IFN-γ-inducible genes, Fig. 2c) performed quite similarly. Method 1 (recognition of the type I IFN-inducible genes) requires improvement because of a high overprediction (Fig. 2b).

### Search of the potential ISGs in the human genome

To minimize the overpredictions admitted in the search of the potential ISGs in the human genome, we applied together all the three methods. This enabled us to detect with high significance the early response ISGs with a several fold enhancement of the expression in the response to IFNs. To determine the number of early response ISGs in the human genome, we used the EPD database [16] because it contains well-documented TSSs. The -1000 to +1000 bp regions of the 1664 human genes annotated in the EPD database were analyzed. The training and control sets were analyzed in the same way. The recognition performance for the ISGs in the various samples is set out in Table 6.

Among the 1664 human genes retrieved from EPD, 74 that potentially responded to the IFN induction were recognized (Table 6). For 60 of these, IFN induction had not been detected. For the 14 other genes, RNA microarray analyses provided experimental support for transcription enhancement by IFN. In 28 of the recognized genes, the -200 to +50 bp promoter regions were the most sensitive to IFN induction.

**Table 6:** Recognition of the ISGs for various samples

Sample	Total number of sequences in a sample	Number of recognized genes	Recognized genes, %
control-EPD set	1664	74	4.4
control-RefSeq set	6809	79	1.2
training-ISG set	72	17	23.6
microarray-ISG set	1004	39	3.9
control-Gluco set	70	0	0
control-LipM set	58	0	0

The detected potential ISGs may be grouped by the IFN biological activities. These groups include immune and inflammatory responses, regulation of cell proliferation and differentiation, antitumor effect (Additional file 3: Putative human ISG recognized by the methods developed in the EPD database). Genes whose involvement in biological functions of IFNs was doubtful were regarded as possible overestimations and they were all assigned to the possible false-positives.

The regulatory regions of the genes of the IFN system have been systematically studied for many years. As a consequence, the number of ISGs with well-annotated TSSs may be relatively high and EPD-set is more enriched in these genes than the RefSeq-set. Thus, our estimates of the ISG number in the human genome may turn out to be much higher than the EPD-based. Given the fact that not all TSSs are well-annotated in the RefSeq, higher underpredictions in RefSeq-based datasets may be expected because our ISG recognition was strongly dependent on the accuracy of TSS detection.

According to our estimates based on EPD and given the possible overprediction (21 genes of 78, 26.9%), the human genome contains about 3000 ISGs. However, the RefSeq-based estimates are more than threefold smaller (~1000 ISGs). It is likely that the real number lies somewhere between these two numbers, *i.e*. from 1500 to 2000 ISGs per human genome. This estimate agrees with the one determined using microarrays. In the primary monocytes from the peripheral blood mononuclear cells from HCV infected patients at least twofold induction during the first 6 hours after IFNα treatment was observed for 1012 genes [13], whereas the number of IFNγ-inducible genes was 632 in macrophages [17]. Our results obtained by method 2 are consistent with the prediction results for the IFNγ-inducible genes: 65% of the predicted genes were, indeed, IFNγ-inducible; 1387 genes out of 13,668 genes were predicted as IFNγ-inducible [18]. In the HepG2 cell line, 400 genes of 14,112 analyzed genes were induced more than twofold by IFNα and 405 by IFNγ [19]. In the HT1080 fibrocarcinoma cell line, over 1000 of 6,800 genes were inducible by the type I IFNs during the first 6 hours of induction, however, a less than twofold induction was observed for 75% of them [14]. The gene sets inducible by different IFNs and in cells of different types do not overlap entirely. Out of 4600 genes examined in IFN-treated HeLa, hepatoma cell lines and primary embryonic hepatocytes, 50 were consistently IFN-inducible, by contrast, the IFN stimulation of another 60 genes was cell-type specific [20].

We believe that further experimental and computational studies will provide a better understanding of the genes involved in IFN induction and ultimately of the molecular mechanisms of this induction, thereby more precise prediction of the side effects of IFN therapy.

## Conclusion

1. Approaches for the recognition of the 20 BSs including BSs for the key regulators of the IFN system, namely, IRF1, ISGF3, STAT1, and NF-κB were developed.

2. Specific combinations of various BSs were revealed for the IFN-inducible regions of the three analyzed types.

3. To provide more efficient tools for ISG recognition, we devised three computer-assisted methods for the prediction of the IFN-inducible regions relying on the increase in the occurrence frequency of BS combinations in the 5'-regions of ISGs.

4. About 200 genes were confidently predicted as ISGs out of 10,000 gene sequences from in the RefSeq, EPD, and TRRD databases.

## Methods

### Samples

The BSs samples of the key regulators (ISGF3, IRF1, STAT1, and NF-κB) were compiled using the information stored in TRRD [21]. Sample size varied in the 25–70 range for the sequences of a particular type. Sequence length was 60 bp. The samples of the other BSs (AP1, C/EBP, E2F, GATA1, GR, HNF1, HNF3, HNF4, MyoD, NF-Y, OCT1, SF1, Sp1, and TATA-box) were partly taken from the TRRD, partly from the SELEX databases [22]. The recognition methods of all the BSs were built as described below. To make the training-ISG set, we chose 73 human genes from the TRRD database for which the IFN induction was confirmed (Additional file 4: Training sample of the human ISGs annotated in the TRRD database).

The sample of human glucocorticoid regulated genes (control-Gluco set) containing 39 promoter sequences was partly compiled from those stored in TRRD, partly from the literary and RefSeq data. The sample of the lipid metabolism genes (control-LipM set) from TRRD was kindly provided by Ignatieva E.V, it contained 59 human promoter sequences. The sequences for the microarray ISG-sets (for the list of articles see Additional file 2: List of references for drawing the ISGs samples from the microarray data), were extracted from the human RefSeq contigs. The control sample of human random promoters (control-RefSeq set) contained 8285 sequences extracted from the human RefSeq contigs. The sequences in all samples, with the exception of the control-EPD set, were 7000 bp long, from -5000 to +2000 bp with respect to TSS. The control-EPD set contained 1664 human sequences 2000 bp in length, from -1000 to +1000 bp with respect to TSS.

The TSS was determined using either the data stored in the TRRD database, or the corresponding mRNA annotated in the RefSeq.

### Building of weight matrices

To recognize the BSs, a matrix approach based on the additive or multiplicative function was used. To construct the frequency and weight matrices, an iterative method was developed.

Building of a weight matrix for the description of the structure of the BSs was based on a detailed analysis of a given sample of **m **nucleotide sequences. It was assumed that each sample sequence of 100 bp contains one and only one BS whose exact location and DNA strand orientation are, however, unknown. To calculate the weight matrix, three multiple alignment methods similar to the Gibbs sampling method, were used. The methods were different in that the recognition function and the procedures for the transformation of the frequency into weight matrices were different. The algorithms of the methods were iterative, each iteration being two-step. To work with the algorithms, an initial approximation to the frequency matrix is assumed F = (f_ij_), i = {A,C,G,T} j = 1,...,*l*, where *l *denotes site length, f_ij _is the occurrence frequency of the nucleotide i at the j-th position of the aligned sample.

At the first iteration step, the frequency matrix F is transformed into the weight matrix W = (w_ij_): W = T(F) by transformation T. The explicit form of the transformation T is given below. Moving along the first sample sequence with 1 bp step, the recognition function value G (the explicit form of the function is given below) is calculated for each analyzed sequence fragment *l *long. The same calculations are done for the reverse strand. The fragment to which the maximum value of the recognition function corresponds was chosen as the sole candidate site. Then, the procedure was applied, one by one, to the other sample sequences so that ultimately **m **candidate sites were detected.

All the **m **candidates detected at step 1 were aligned taking into account the strand orientation. A new frequency matrix was calculated for the obtained alignment. When the matrix perfectly matched with the preceding F version, iteration was over. This ended up in an aligned site sample and a weight matrix corresponding to it.

The three methods used the following versions of the recognition function G and of the transformation T of the frequency to the weight matrix:

### Method 1

T: w_ij _= f_ij_/(f_Aj _+ f_Cj _+ f_Gj _+ f_Tj_),     (1)

For the given S = s_1_,...,*s*_*l *_nucleotide sequence *l *long, the recognition function G is calculated using the additive function (2)

G(s1,...,sl)=∑i=1,...,lWsii.     (2)
 MathType@MTEF@5@5@+=feaafiart1ev1aaatCvAUfKttLearuWrP9MDH5MBPbIqV92AaeXatLxBI9gBaebbnrfifHhDYfgasaacH8akY=wiFfYdH8Gipec8Eeeu0xXdbba9frFj0=OqFfea0dXdd9vqai=hGuQ8kuc9pgc9s8qqaq=dirpe0xb9q8qiLsFr0=vr0=vr0dc8meaabaqaciaacaGaaeqabaqabeGadaaakeaacqqGhbWrcqGGOaakcqqGZbWCdaWgaaWcbaGaeGymaedabeaakiabcYcaSiabc6caUiabc6caUiabc6caUiabcYcaSiabbohaZnaaBaaaleaacqWGSbaBaeqaaOGaeiykaKIaeyypa0ZaaabuaeaacqWGxbWvdaWgaaWcbaGaem4Cam3aaSbaaWqaaiabdMgaPbqabaaaleqaaOGaemyAaKgaleaacqWGPbqAcqGH9aqpcqaIXaqmcqGGSaalcqGGUaGlcqGGUaGlcqGGUaGlcqGGSaalcqWGSbaBaeqaniabggHiLdGccqGGUaGlcaWLjaGaaCzcamaabmaabaGaeGOmaidacaGLOaGaayzkaaaaaa@5034@

### Method 2

The transformation is T as in method 1. The G function for the sequence S is calculated using the multiplicative function

G(s1,...,sl)=∏i=1,...,lWsii
 MathType@MTEF@5@5@+=feaafiart1ev1aaatCvAUfKttLearuWrP9MDH5MBPbIqV92AaeXatLxBI9gBaebbnrfifHhDYfgasaacH8akY=wiFfYdH8Gipec8Eeeu0xXdbba9frFj0=OqFfea0dXdd9vqai=hGuQ8kuc9pgc9s8qqaq=dirpe0xb9q8qiLsFr0=vr0=vr0dc8meaabaqaciaacaGaaeqabaqabeGadaaakeaacqqGhbWrcqGGOaakcqqGZbWCdaWgaaWcbaGaeGymaedabeaakiabcYcaSiabc6caUiabc6caUiabc6caUiabcYcaSiabdohaZnaaBaaaleaacqWGSbaBaeqaaOGaeiykaKIaeyypa0ZaaebuaeaacqWGxbWvdaWgaaWcbaGaem4Cam3aaSbaaWqaaiabdMgaPbqabaaaleqaaaqaaiabdMgaPjabg2da9iabigdaXiabcYcaSiabc6caUiabc6caUiabc6caUiabcYcaSiabdYgaSbqab0Gaey4dIunakiabdMgaPbaa@4B6D@

### Method 3

The calculation of the weight matrix is two-step. First, the transformation used in method 1 is applied, next, the entropy E_j _is calculated for each position j, j = 1,...,*l *using formula (3)

Ej=−∑i=A,C,G,Twij×ln⁡(wij)     (3)
 MathType@MTEF@5@5@+=feaafiart1ev1aaatCvAUfKttLearuWrP9MDH5MBPbIqV92AaeXatLxBI9gBaebbnrfifHhDYfgasaacH8akY=wiFfYdH8Gipec8Eeeu0xXdbba9frFj0=OqFfea0dXdd9vqai=hGuQ8kuc9pgc9s8qqaq=dirpe0xb9q8qiLsFr0=vr0=vr0dc8meaabaqaciaacaGaaeqabaqabeGadaaakeaacqqGfbqrdaWgaaWcbaGaeeOAaOgabeaakiabg2da9iabgkHiTmaaqafabaGaee4DaC3aaSbaaSqaaiabbMgaPjabbQgaQbqabaaabaGaemyAaKMaeyypa0JaemyqaeKaeiilaWIaem4qamKaeiilaWIaem4raCKaeiilaWIaemivaqfabeqdcqGHris5aOGaey41aqRagiiBaWMaeiOBa4MaeiikaGIaee4DaC3aaSbaaSqaaiabbMgaPjabbQgaQbqabaGccqGGPaqkcaWLjaGaaCzcamaabmaabaGaeG4mamdacaGLOaGaayzkaaaaaa@4FCA@

The final weights w_ij_* are obtained by renormalization of the initial weights w_ij _by applying formula (4)

w_ij_* = w_ij_/E_j_*,     (4),

where E_j_* is the modified entropy at the j^th ^position, i.e.

E_j_* = {E_j_, if E_ji _> 0.1; 0.1 *otherwise*}.

To calculate the recognition function, the same additive function (2) as in method 1 is used.

Three matrices result from treatment of the same sample sites with the three methods. The matrices do not match, as a rule, yet they are very similar. Comparative analysis of each of the 3 built matrices allows to choose the final matrix as the one that provides the smallest recognition error of type II (overprediction, α_2_) at a fixed error of type I α_1 _= 15%.

It should be noted that the methods described above for the derivation of the weight matrices are partly based on the same principles as the Gibbs sampler approach [23] for multiple alignment. Both assume that there exists one and only one site in every nucleotide sequence. In both cases, the methods are iterative, two-step, closely related to the Estimation-Maximization technique. There is an essential difference between the two, however. Gibbs sampler approach relies on conditional probabilities within the framework of the Bayesian model. The method we propose for the matrix building is not based on the probability model. With our method, we optimize the sum of the scores assigned to the BSs in the training-ISG set.

### Method of statistical simulation

To reduce errors of type II, the method of statistical simulation was utilized. If recognition function value was above the threshold, a greater number (~10^7^) of 500 bp random sequences with given nucleotide frequencies was additionally simulated. Nucleotide frequencies were preliminarily calculated in a 500 bp fragment of the analyzed sequence in whose center the predicted site was located. The α_2 _value was obtained by counting the number of site predictions detected in the simulated random sequences. The final decision making was based on comparison of the calculated error α_2 _with the additional threshold value α_2_*;α_2_* for each site calculated by analysis of the training sample of the corresponding site. In the process of choice of the α_2_* values, the false prediction was minimized provided that error of type I (α_1_) did not increase significantly.

Error of type I (α_1_) was calculated on the training sample by the 4-fold cross-validation technique. Thus, we fitted the matrix model to the 75% training set of BSs and estimated type I error of the fitted model on the remaining part (25%) of the training set. Type II error was estimated by statistical simulation. To do so, we calculated the nucleotide frequency for each sequence from the control-EPD set. Then, using the calculated frequencies, the corresponding random sequence was simulated. This was done under the assumption that the matrix approach is based on the principle of position independence. Finally, type II error was estimated as the relative site frequency in all the simulated sequences.

Table 7 gives the values of type I and type II errors (α_1 _and α_2, _respectively) and also the underestimation values for the additional control samples (the last column), which contained the site sequences initially not included in the training samples. For most BSs included in the additional control samples, binding of the TFs was confirmed only indirectly by cross-competition in gel-shift assays.

**Table 7:** Accuracy of the BS recognition

Binding site for the TF	Type I error (α_1_) underprediction	Type II error (α_2_) overprediction	Independent control (underestimation value at the given α_2_)
IRF1	24%	9.59E-05	31.8%
ISGF3	25%	6.84E-04	46.2%
NF-κ B	42%	5.32E-04	70.8%
STAT1	43%	8.82E-05	84.6%

### Building of recognition methods for the interferon-inducible promoters and enhancers

In building the methods, we analyzed, in a given region, the occurrence frequencies of key regulator BSs (ISGF3, IRF1, STAT1, and NF-κB) and combinations of these key regulator BSs with each other and with the sites of the other types, namely, AP1, GATA1, OCT1, Sp1, and TATA-box. Comparison of the training-ISG set with the control-EPD set was decisive in derivation of the statistically significant patterns (individual sites or site pairs).

Only those patterns whose occurrence frequencies in the training-ISG set significantly exceeded those in the control set were selected. To identify individual patterns, the gene regulatory regions from the training and control sets were examined. The regions were of different lengths (not shorter than 200 bp) and differently disposed relative to the TSS. Many of the regions overlapped. A site or a site pair was accepted as a statistically significant pattern, if at least one of the examined regions was significant. When several, as a rule, overlapping regions were significant at the same time, the region with the greatest significance was given preference.

In selection of individual sites, the statistical test of comparison of two binomial variables was applied. With this test, the site is classified as a pattern, if its relative occurrence frequency in the training-ISG set (F1) significantly exceeded that in the control-EPD set (F2). Here "relative" meant that the frequencies were normalized and lay in the 0–1 range. The maximally accepted p-value was 0.01. The standard χ^2 ^test based on the 2 × 2-contingency tables was used initially for identification of site pairs within the training-ISG set (p-value ≤ 0.01). In fact, the reached p-values ≤ 0.001 for most of the chosen site pairs, thereby supporting the high statistical significance of the results. Then, only the identified site pairs with occurrence frequency higher in the training-ISG set than in the control-EPD set were further regarded as patterns.

After selecting the significant patterns, the score at any position (say, *pos_fixed*) of an arbitrary nucleotide sequence SEQ0 was calculated using the following algorithm. Let T_1_, T_2_,..., T_m _be the **m **patterns identified on the basis of analysis of the training-ISG set. Then, the **m **weights w_1_, w_2_, ..., w_m _were calculated for fixed position *pos_fixed *of the sequence SEQ0:

w_i _= {1, if the i-th pattern T_i _is not present at the corresponding positions with respect to the fixed position *pos_fixed*;

W_i _= F1/F2, if the i-th pattern T_i _is present at the corresponding positions with respect to the fixed position *pos_fixed*}, i = 1,...,m.

It should be noted that selected patterns were individual sites or their pairs at defined distances from the TSS. However, in calculation of the w_i_, i = 1,...,m, position *pos_fixed*, the presence of the same sites or site pairs equidistant from the fixed position pos_fixed in the SEQ0 sequence was checked. The multiplicative function was used to calculate the SCORE for a given *pos_fixed *position of the SEQ0.

SCORE = w_1 _* w_2 _* ... * w_m_.     (5)

The function that measures the similarity between the examined SEQ0 sequence and the training-ISG set from which the T_1_, T_2_, ..., T_m _patterns were derived. This is followed by the scoring of all the positions of the SEQ0. The position with the greatest score was ultimately selected. Each method uses its own set of selected patterns T_1_, T_2_, ..., T_m_.

In analysis of an arbitrary sequence, the score was calculated depending on the selected patterns. The score is calculated by using the multiplicative function (5). A score was assigned to the prediction by each method.

SCORE1, the ISG of the general type (method 0)

SCORE2, the IFNγ-inducible regions (method 2)

SCORE3, the IFNα/β-inducible regions (method 1)

## Abbreviations

bp, base pair;

BS, binding site;

IFN, interferon;

IRF, IFN regulatory factor;

ISG, IFN-stimulated genes;

ISGF, IFN-stimulated gene factor;

STAT, signal transducer and activator of transcription;

TF, transcription factor;

TSS, transcription start site.

## Authors' contributions

EAA conceived of the study, and participated in the setting up of the training samples, determination of the threshold values and adjustment of methods; estimation of the biological significance of the recognition results and drafted the manuscript; biological background.

JuVK participated in the sequence alignment, development of all the recognition methods; determination of the threshold values and adjustment of methods; determination of the statistical significance of the results and drafted the manuscript; mathematical and statistical background.

TIM participated in the design of the study, setting up of the sample of the glucocorticoid-regulated genes; estimation of the reliability of the recognition results; biological background.

NAK participated in the design and coordination of the study and helped to draft the manuscript.

All authors read and approved the final manuscript.

## Supplementary Material

Additional File 1 **Distribution of IRF1, ISGF3, STAT1, and NF-κB binding sites in different gene groups**. The diagrams of the four binding sites distribution in the promoter regions (from -500 to +500 with respect to the transcriptional start site) of IFN-inducible, glucocorticoid-regulated, and genes of lipid metabolism. Click here for file

Additional File 2 **List of references for drawing the ISGs samples from microarray data**. References to the sources of the data for the microarray-ISG sets. Click here for file

Additional File 3 **Putative human ISG recognized by the methods developed in the EPD database**. The table contains gene names, recognition function values, maximum of the recognition function (position with respect to the TSS). Click here for file

Additional File 4 **Training sample of the human ISGs annotated in the TRRD database**. The table contains gene names, TRRD accession numbers, and information about the gene inducibility by IFNs. Click here for file
