# Supplementary material for: Recognition of interferon-inducible sites, promoters, and enhancers
Source: BMC Bioinformatics. 2007 Feb 19;8:56. doi: 10.1186/1471-2105-8-56 (PMC1810324; doi:10.1186/1471-2105-8-56)
Supplement: Additional File 1 — Distribution of IRF1, ISGF3, STAT1, and NF-κB binding sites in different gene groups. The diagrams of the four binding sites distribution in the promoter regions (from -500 to +500 with respect to the transcriptional start site) of IFN-inducible, glucocorticoid-regulated, and genes of lipid metabolism. [file 1471-2105-8-56-S1.pdf]

Distribution of IRF1, ISGF3, STAT1, and NF-κB binding sites in different gene groups

X axis, position with respect to the transcription start site
Y axis, occurrence frequency of putative binding sites

Legend:
Glucocorticoid-regulated genes, blue
Genes of lipid metabolism, yellow
IFN-inducible genes, green

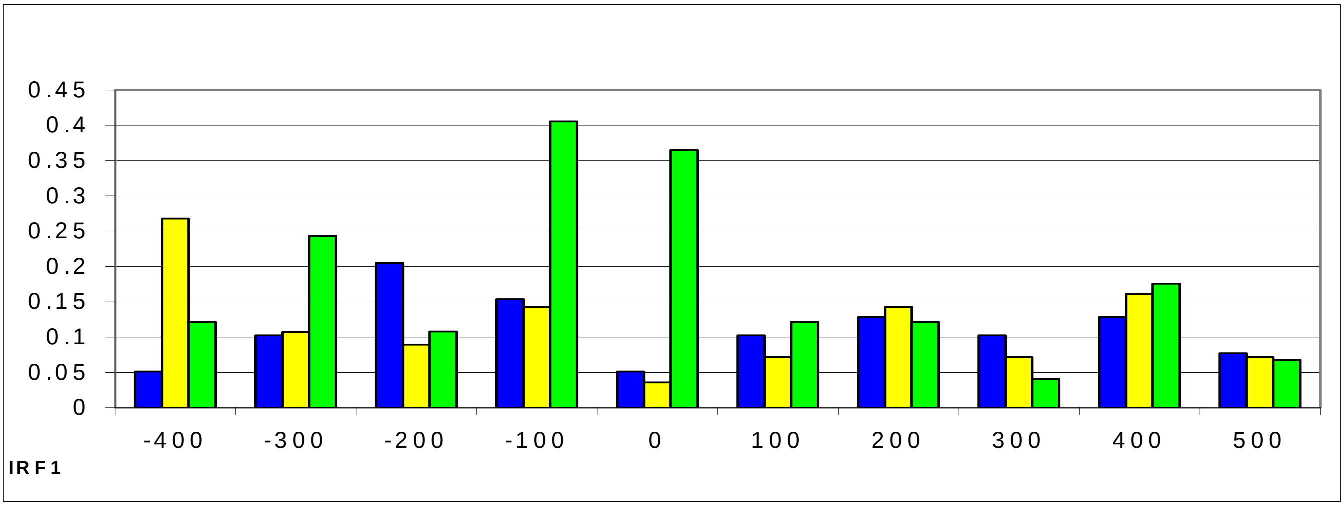

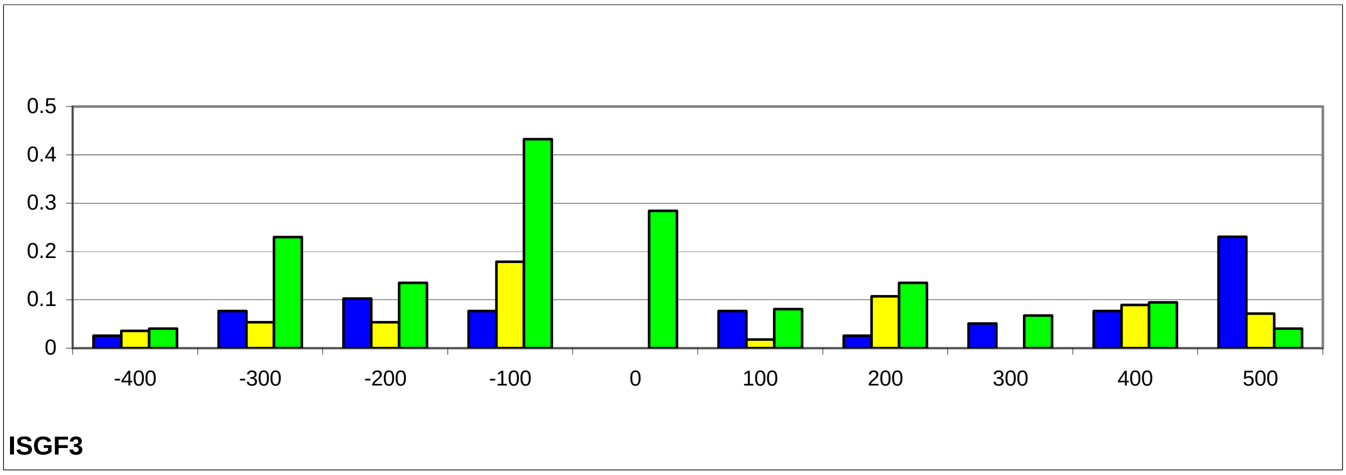

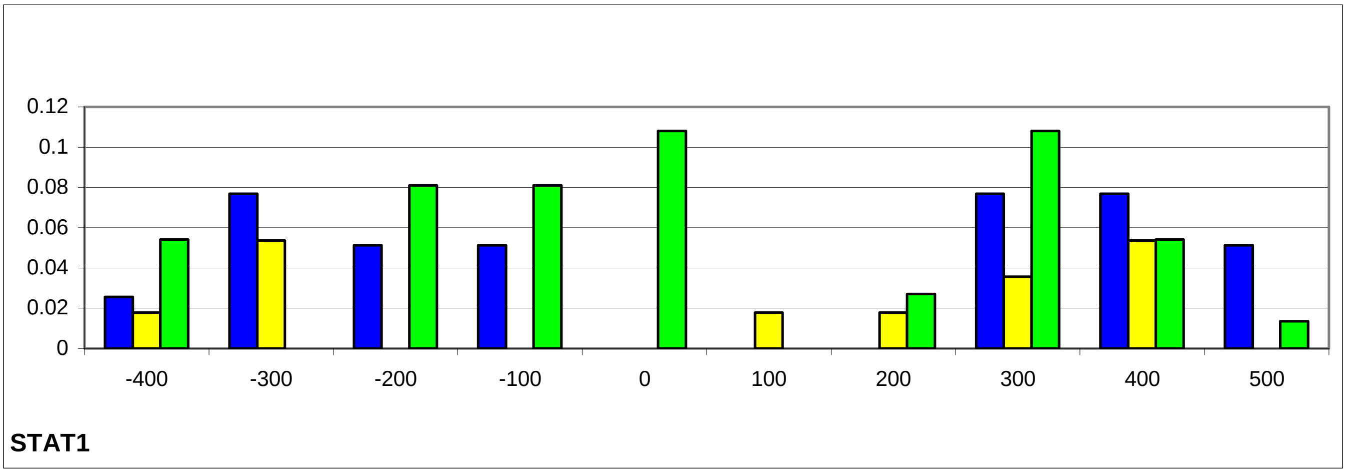

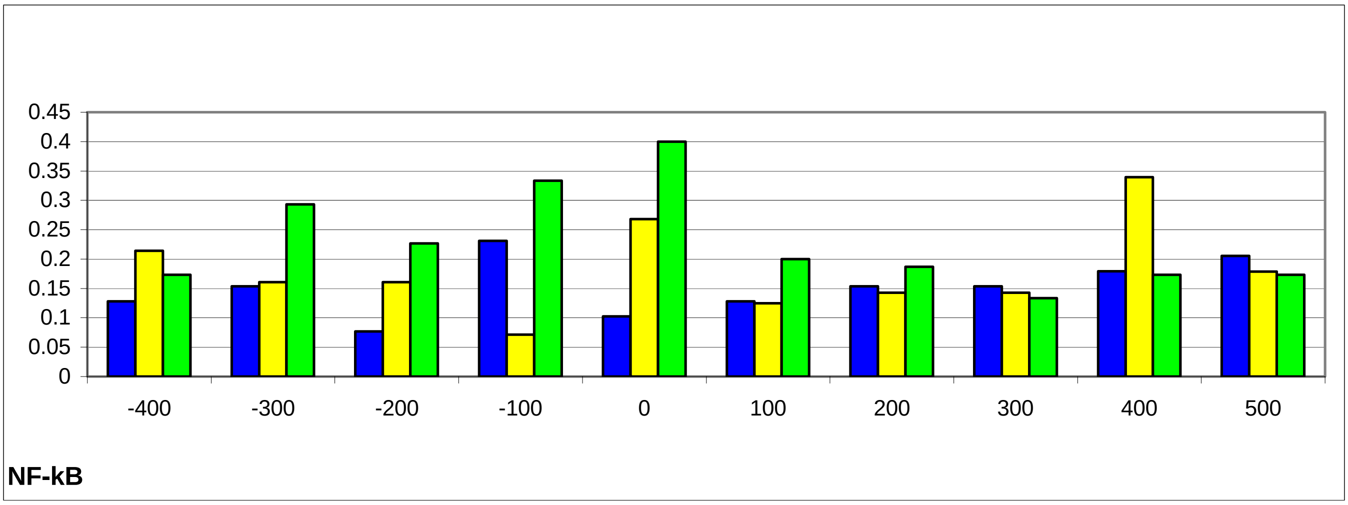

0.45
0.4
0.35
0.3
0.25
0.2
0.15
0.1
0.05
0
-400
-300
-200
-100
0
100
200
300
400
500
NF-kB
