# Supplementary material for: Recognition of interferon-inducible sites, promoters, and enhancers
Source: BMC Bioinformatics. 2007 Feb 19;8:56. doi: 10.1186/1471-2105-8-56 (PMC1810324; doi:10.1186/1471-2105-8-56)
Supplement: Additional File 2 — List of references for drawing the ISGs samples from microarray data. References to the sources of the data for the microarray-ISG sets. [file 1471-2105-8-56-S2.doc]

### **List of references for drawing the IS**Gs samples from microarray data

Benimetskaya L, Wittenberger T, Stein CA, Hofmann HP, Weller C, Lai JC, Miller P, Gekeler V.
Changes in gene expression induced by phosphorothioate oligodeoxynucleotides (including G3139) in PC3 prostate carcinoma cells are recapitulated at least in part by treatment with interferon-beta and -gamma.

Clin Cancer Res. 2004 Jun 1;10(11):3678-88.

PMID: 15173074

Der SD, Zhou A, Williams BR, Silverman RH.

Identification of genes differentially regulated by interferon alpha, beta, or gamma using oligonucleotide arrays.

Proc Natl Acad Sci U S A. 1998 Dec 22;95(26):15623-8.

PMID: 9861020

Ehrt S, Schnappinger D, Bekiranov S, Drenkow J, Shi S, Gingeras TR, Gaasterland T, Schoolnik G, Nathan C.

Reprogramming of the macrophage transcriptome in response to interferon-gamma and Mycobacterium tuberculosis: signaling roles of nitric oxide synthase-2 and phagocyte oxidase.

J Exp Med. 2001 Oct 15;194(8):1123-40.

PMID: 11602641

Geiss GK, Carter VS, He Y, Kwieciszewski BK, Holzman T, Korth MJ, Lazaro CA, Fausto N, Bumgarner RE, Katze MG.

Gene expression profiling of the cellular transcriptional network regulated by alpha/beta interferon and its partial attenuation by the hepatitis C virus nonstructural 5A protein.

J Virol. 2003 Jun;77(11):6367-75.

PMID: 12743294

Girard S, Shalhoub P, Lescure P, Sabile A, Misek DE, Hanash S, Brechot C, Beretta L.

An altered cellular response to interferon and up-regulation of interleukin-8 induced by the hepatitis C viral protein NS5A uncovered by microarray analysis.

Virology. 2002 Apr 10;295(2):272-83.

PMID: 12033786

Gomez D, Reich NC.

Stimulation of primary human endothelial cell proliferation by IFN.

J Immunol. 2003 Jun 1;170(11):5373-81.

PMID: 12759411

Grandvaux N, Servant MJ, tenOever B, Sen GC, Balachandran S, Barber GN, Lin R, Hiscott J.

Transcriptional profiling of interferon regulatory factor 3 target genes: direct involvement in the regulation of interferon-stimulated genes.

J Virol. 2002 Jun;76(11):5532-9.

PMID: 11991981

Ji X, Cheung R, Cooper S, Li Q, Greenberg HB, He XS.

Interferon alfa regulated gene expression in patients initiating interferon treatment for chronic hepatitis C.

Hepatology. 2003 Mar;37(3):610-21. Erratum in: Hepatology. 2003 Jun;37(6):1503.

PMID: 12601359

Khabar KS, Al-Haj L, Al-Zoghaibi F, Marie M, Dhalla M, Polyak SJ, Williams BR.

Expressed gene clusters associated with cellular sensitivity and resistance towards anti-viral and anti-proliferative actions of interferon.

J Mol Biol. 2004 Sep 17;342(3):833-46.

PMID: 15342240

Mossman KL, Macgregor PF, Rozmus JJ, Goryachev AB, Edwards AM, Smiley JR.

Herpes simplex virus triggers and then disarms a host antiviral response.

J Virol. 2001 Jan;75(2):750-8.

PMID: 11134288

Presti RM, Popkin DL, Connick M, Paetzold S, Virgin HW 4th.

Novel cell type-specific antiviral mechanism of interferon gamma action in macrophages.

J Exp Med. 2001 Feb 19;193(4):483-96.

PMID: 11181700

Urosevic M, Oberholzer PA, Maier T, Hafner J, Laine E, Slade H, Benninghoff B, Burg G, Dummer R.

Imiquimod treatment induces expression of opioid growth factor receptor: a novel tumor antigen induced by interferon-alpha?

Clin Cancer Res. 2004 Aug 1;10(15):4959-70.

PMID: 15297396

de Veer MJ, Holko M, Frevel M, Walker E, Der S, Paranjape JM, Silverman RH, Williams BR.

Functional classification of interferon-stimulated genes identified using microarrays.

J Leukoc Biol. 2001 Jun;69(6):912-20. Review.

PMID: 11404376

Wang X, Yuan ZH, Zheng LJ, Yu F, Xiong W, Liu JX, Hu GX, Li Y.

Gene expression profiles in an hepatitis B virus transfected hepatoblastoma cell line and differentially regulated gene expression by interferon-alpha.

World J Gastroenterol. 2004 Jun 15;10(12):1740-5.

PMID: 15188497

Xiong W, Wang X, Liu XY, Xiang L, Zheng LJ, Liu JX, Yuan ZH.

Analysis of gene expression in hepatitis B virus transfected cell line induced by interferon.

Sheng Wu Hua Xue Yu Sheng Wu Wu Li Xue Bao (Shanghai). 2003 Dec;35(12):1053-60.

PMID: 14673494

Yan W, Lee H, Yi EC, Reiss D, Shannon P, Kwieciszewski BK, Coito C, Li XJ, Keller A, Eng J, Galitski T, Goodlett DR, Aebersold R, Katze MG.

System-based proteomic analysis of the interferon response in human liver cells.

Genome Biol. 2004;5(8):R54. Epub 2004 Jul 22.

PMID: 15287976
