# Supplementary material for: Recognition of interferon-inducible sites, promoters, and enhancers
Source: BMC Bioinformatics. 2007 Feb 19;8:56. doi: 10.1186/1471-2105-8-56 (PMC1810324; doi:10.1186/1471-2105-8-56)
Supplement: Additional File 3 — Putative human ISG recognized by the methods developed in the EPD database. The table contains gene names, recognition function values, maximum of the recognition function (position with respect to the TSS). [file 1471-2105-8-56-S3.doc]

### **Putative human ISG recognized by the methods developed in the EPD database**

| **Immune and inflammatory responses (20)** | | | | | | |
| --- | --- | --- | --- | --- | --- | --- |
| Gene name | Recognition function value (Method0/Method1/ Method 2) | | Maximum of the recognition function (with respect to the TSS) | | Biological function of the encoded protein | |
| *BLMH* | 0.73 / 0.37 / 0.47 | | 9 | | Generation of antigenic peptides presented to MHC class I molecules | |
| *LGMN* | 0.43 / 0.10 / 0.70 | | 250 | | Processing of proteins for MHC class II antigen presentation in the lysosomal/endosomal system | |
| *PSMB10* | 0.50 / 0.66 / 0.66 | | -2 | | Antigen processing to generate class I binding peptides | |
| *DUT* | 0.67 / 0.46 / 0.48 | | -7 | | Nucleotide metabolism, a potential antiparasitic. | |
| *ADA* | 0.58 / 0.62 / 0.24 | | -122 | | Antimicrobial humoral response | |
| *PTMA* | 0.44 / 0.23 / 0.80 | | 147 | | Resistance to certain opportunistic infections | |
| *SNRPD3* | 0.41 / 0.41 / 0.42 | | 405 | | Enhanced expression during autoimmune diseases | |
| *M6PR* | 0.48 / 0.10 / 0.48 | | 62 | | Transport of phosphorylated lysosomal enzymes from the Golgi complex and the cell surface to lysosomes, receptor mediated endocytosis | |
| *TUBB2A* | 0.41 / 0.62 / 0.12 | | 300 | | MHC class I protein binding, natural killer cell mediated cytotoxicity | |
| *BTF3* | 0.43 / 0.55 / 0.24 | | 177 | | Immunoglobulin superfamily | |
| *LGALS3* | 0.49 / 0.62 / 0.34 | | 281 | | Galactose-specific lectin that binds IgE | |
| *TSPAN7* | 0.46 / 0.32 / 0.54 | | 378 | | Proliferation and cell motility of T-cells | |
| *HIF1A* | 0.65 / 0.35 / 0.57 | | -263 | | Response to hypoxia, angiogenesis | |
| *PSMB3* | 0.40 / 0.16 / 0.62 | | -334 | | ATP-dependent proteolytic activity | |
| *PSMC6* | 0.63 / 0.31 / 0.98 | | 260 | | ATP-dependent degradation of ubiquitinated proteins | |
| *PSMD1* | 0.41 / 0.58 / 0.62 | | -406 | | ATP-dependent degradation of ubiquitinated proteins | |
| *UBE2D1* | 0.55 / 0.12 / 0.58 | | -26 | | Selective degradation of short-lived and abnormal proteins. | |
| *SRP54* | 0.42 / 0.28 / 0.87 | | -132 | | Binds to the signal sequence of presecretory protein when they emerge from the ribosomes and transfers them to TRAM | |
| *FOSL1* | 0.49 / 0.62 / 0.18 | | 102 | | Transcription factor, cellular defense response | |
| *JUN* | 0.52 / 0.11 / 0.62 | | 169 | | Transcription factor | |
| **Regulation of proliferation (3)** | | | | | | |
| Gene name | | Recognition function value (Method0/Method1/ Method 2) | | Maximum of the recognition function (with respect to the TSS) | | Biological function of the encoded protein |
| *BUB3* | | 0.70 / 0.19 / 0.62 | | -139 | | Mitotic checkpoint proteins act as transcriptional repressors during interphase |
| *KHDRBS1* | | 0.43 / 0.23 / 0.66 | | -54 | | Cell cycle arrest |
| *RBBP4* | | 0.56 / 0.24 / 0.59 | | -172 | | Negative regulation of cell proliferation. Chromatin assembly in DNA replication and DNA repair |
| **Regulation of cell differentiation (24)** | | | | | | |
| Gene name | | Recognition function value (Method0/Method1/ Method 2) | | Maximum of the recognition function (with respect to the TSS) | | Biological function of the encoded protein |
| *COPE* | | 0.40 / 0.20 / 0.77 | | 249 | | Intra-Golgi transport; processing, activity, and endocytic recycling of LDL receptors |
| *CFL1* | | 0.40 / 0.81 / 0.21 | | -269 | | Actin cytoskeleton organization and biogenesis, Rho protein signal transduction |
| *CTNNA2* | | 0.53 / 0.64 / 0.33 | | -158 | | Cytoskeleton formation |
| *HNRPR* | | 0.87 / 0.73 / 0.62 | | 167 | | Processing of precursor mRNA |
| *PPIF* | | 0.81 / 0.62 / 0.55 | | 73 | | Folding of proteins |
| *CCT6A* | | 0.64 / 0.27 / 0.50 | | 70 | | Folding of proteins in an ATP-dependent fashion |
| *TPI1* | | 0.41 / 0.73 / 0.14 | | 71 | | Plays an important role in several metabolic pathways |
| *SLC38A2* | | 0.65 / 0.46 / 0.58 | | 251 | | Amino acid transport |
| *TM9SF2* | | 0.61 / 0.19 / 0.52 | | 127 | | Integral membrane protein may function as a channel or small molecule transporter |
| *ARF1* | | 0.58 / 0.79 / 0.27 | | -225 | | Protein trafficking |
| *CD63* | | 0.48 / 0.56 / 0.57 | | -188 | | Growth regulation, lysosomal membrane protein |
| *ADFP* | | 0.46 / 0.25 / 0.68 | | -402 | | Adipose differentiation-related protein |
| *GNG5* | | 0.49 / 0.40 / 0.93 | | -407 | | Modulator or transducer in various transmembrane signaling systems |
| *GNPDA1* | | 0.77 / 0.48 / 0.44 | | 329 | | Generation of precursor metabolites and energy |
| *SUCLG1* | | 0.58 / 0.48 / 0.98 | | -70 | | Succinyl-CoA synthetase mitochondrial |
| *TFAM* | | 0.48 / 0.17 / 0.43 | | -115 | | Mitochondrial transcription regulation |
| *GOT2* | | 0.51 / 0.27 / 0.54 | | 157 | | Aspartate catabolism, mitochondrion |
| *NDUFA9* | | 0.62 / 0.38 / 0.55 | | -314 | | Transfer of electrons from NADH to the respiratory chain |
| *PHGDH* | | 0.51 / 0.08 / 0.50 | | 440 | | Electron transporter activity |
| *RPS8* | | 0.63 / 0.23 / 0.55 | | 166 | | 40S ribosomal protein S8 |
| *RPL37* | | 0.54 / 0.32 / 0.70 | | -448 | | 60S ribosomal protein L37 |
| *EIF3S3* | | 0.46 / 0.12 / 0.55 | | 10 | | Translation initiation |
| *EIF3S12* | | 0.48 / 0.21 / 0.46 | | 63 | | Translation initiation |
| *EIF2S2* | | 0.51 / 0.51 / 0.35 | | 77 | | Translation initiation |
| **Suppression of tumors (2)** | | | | | | |
| Gene name | | Recognition function value (Method0/Method1/ Method 2) | | Maximum of the recognition function (with respect to the TSS) | | Biological function of the encoded protein |
| *RPL10* | | 0.59 / 0.11 / 0.53 | | -316 | | Tumor suppressor |
| *RUVBL2* | | 0.56 / 0.00 / 0.67 | | -12 | | Tumor suppressor mediated growth arrest and replicative senescence, apoptosis, DNA repair |
| **Unknown interferon function (4)** | | | | | | |
| Gene name | | Recognition function value (Method0/Method1/ Method 2) | | Maximum of the recognition function (with respect to the TSS) | | Biological function of the encoded protein |
| *C14orf92* | | 0.61 / 0.22 / 0.94 | | 399 | | Epidermal Langerhans cell protein LCP1 |
| *IFI27* | | 0.42 / 0.31 / 0.83 | | -81 | | Integral membrane protein, Interferon-alpha-induced |
| *NDUFB7* | | 0.45 / 0.19 / 0.72 | | -162 | | Transfer of electrons from NADH to the respiratory chain, cell adhesion protein |
| *AGR2* | | 0.40 / 0.17 / 0.52 | | 303 | | Secreted (probable) |
| **Possible false-positives (21)** | | | | | | |
| Gene name | | Recognition function value (Method0/Method1/ Method 2) | | Maximum of the recognition function (with respect to the TSS) | | Biological function of the encoded protein |
| *CDIPT* | | 0.71 / 0.46 / 0.82 | | 9 | | Phosphatidylinositol transporter activity |
| *ODC1* | | 0.71 / 0.21 / 0.44 | | -447 | | Polyamine biosynthesis |
| *OAZ1* | | 0.71 / 0.32 / 0.48 | | -147 | | Polyamine biosynthesis |
| *GGPS1* | | 0.47 / 0.40 / 0.73 | | -155 | | Isoprenoid biosynthesis |
| *B3GNT6* | | 0.49 / 0.12 / 0.44 | | -363 | | Poly-N-acetyllactosamine biosynthesis |
| *RBM7* | | 0.60 / 0.51 / 0.75 | | 222 | | Possible involved in germ cell RNA processing and meiosis |
| *ZWINT* | | 0.53 / 0.39 / 0.56 | | -317 | | Kinetochore formation and proper chromosome segregation during mitosis |
| *MYCN* | | 0.42 / 0.43 / 0.48 | | -69 | | Transcription factor, proto-oncogene |
| *TPM1* | | 0.43 / 0.21 / 0.64 | | 409 | | Striated muscle contraction |
| *CNP* | | 0.43 / 0.45 / 0.54 | | 287 | | Synaptic transmission brain white matter |
| *TIMM8A* | | 0.53 / 0.21 / 0.54 | | 254 | | Nervous system development |
| *THY1* | | 0.48 / 0.25 / 0.42 | | -351 | | Cell-cell or cell-ligand interactions during synaptogenesis |
| *NPPA* | | 0.48 / 0.23 / 0.57 | | 340 | | Regulation of blood pressure |
| *BAG1* | | 0.49 / 0.26 / 0.47 | | -395 | | Anti-apoptotic activity |
| *SERF2* | | 0.51 / 0.35 / 0.32 | | 104 | | Unknown function. Gastric cancer-related protein |
| *PPP1CA* | | 0.51 / 0.00 / 0.80 | | -401 | | Cell division, glycogen metabolism |
| *LOC57862* | | 0.66 / 0.62 / 0.24 | | 51 | | Unknown function |
| *FLJ10856* | | 0.40 / 0.21 / 0.68 | | -360 | | Unknown function |
| *C6orf37* | | 0.41 / 0.45 / 0.77 | | -276 | | Unknown function |
| *FLJ10432* | | 0.48 / 0.21 / 0.43 | | -135 | | Unknown function |
| *LR8* | | 0.49 / 0.62 / 0.08 | | -92 | | Unknown function |
