# Supplementary material for: Recognition of interferon-inducible sites, promoters, and enhancers
Source: BMC Bioinformatics. 2007 Feb 19;8:56. doi: 10.1186/1471-2105-8-56 (PMC1810324; doi:10.1186/1471-2105-8-56)
Supplement: Additional File 4 — Training sample of the human ISGs annotated in the TRRD database. The table contains gene names, TRRD accession numbers, and information about the gene inducibility by IFNs. [file 1471-2105-8-56-S4.doc]

### Training sample of the human ISGs annotated in the TRRD database

| Gene | TRRD accession number | Inducibility by IFNs |
| --- | --- | --- |
| *BF* | A00341 | IFNγ |
| *CCL11* | A02212 | IFNα/β |
| *CCL13* | A02210 | IFNγ |
| *CCL2* | A00892 | IFNγ |
| *CCL3* | A00989 | IFNγ |
| *CCL5* | A01781 | IFNα/β, IFNγ |
| *CCL7* | A02211 | IFNγ |
| *CD74* | A00275 | IFNγ |
| *CDKN1A* | A00359 | IFNγ |
| *CEACAM1* | A00748 | IFNγ |
| *CXCL10* | A00927 | IFNα/β, IFNγ |
| *CXCL11* | A01148 | IFNα/β |
| *CYBB* | A00991 | IFNγ |
| *FCGR1A* | A00199 | IFNγ |
| *FCGR3B* | A00796 | IFNγ |
| *G1P2* | A00278 | IFNα/β, IFNγ |
| *G1P3* | A00270 | IFNα/β, IFNγ |
| *GBP1* | A00200 | IFNα/β, IFNγ |
| *GRIM19* | A01518 | IFNα/β |
| *HLA-A* | A00186 | IFNα/β, IFNγ |
| *HLA-B* | A00185 | IFNα/β, IFNγ |
| *HLA-DPA1* | A00204 | IFNγ |
| *HLA-DPB1* | A00205 | IFNγ |
| *HLA-DQA1* | A00206 | IFNγ |
| *HLA-DQB1* | A00207 | IFNγ |
| *HLA-DRA* | A00077 | IFNγ |
| *ICAM1* | A00302 | IFNγ |
| *IFI16* | A00902 | IFNγ |
| *IFI44* | A00340 | IFNα/β |
| *IFIT1* | A00272 | IFNα/β, IFNγ |
| *IFIT2* | A00279 | IFNα/β, IFNγ |
| *IFITM1* | A00271 | IFNα/β I, IFNγ |
| *IFNA1* | A00273 | IFNα/β |
| *IFNG* | A00375 | IFNα/β |
| *IL12A* | A02231 | IFNγ |
| *IL12B* | A02232 | IFNγ |
| *IL1B* | A00113 | IFNγ |
| *IL2RA* | A00173 | IFNγ |
| *IL2RB* | A00181 | IFNγ |
| *IL6* | A00115 | IFNγ |
| *IL8* | A00038 | IFNα/β, IFNγ |
| *INDO* | A00358 | IFNα/β, IFNγ |
| *IRF1* | A00276 | IFNα/β, IFNγ |
| *IRF2* | A00277 | IFNα/β, IFNγ |
| *IRF7* | A01777 | IFNα/β |
| *IVL* | A02122 | IFNγ |
| *KRT17* | A00333 | IFNγ |
| *LGALS3BP* | A01070 | IFNα/β |
| *LY6E* | A00280 | IFNα/β, IFNγ |
| *MHC2TA* | A01150 | IFNγ |
| *MMP3* | A00214 | IFNα/β |
| *MT2A* | A00071 | IFNα/β, IFNγ |
| *MX1* | A00703 | IFNα/β |
| *NCF1* | A00731 | IFNγ |
| *NCF2* | A00993 | IFNγ |
| *NFKB1* | A00117 | IFNγ |
| *NOS2A* | A01197 | IFNγ |
| *OAS1* | A00269 | IFNα/β, IFNγ |
| *PIGR* | A01082 | IFNγ |
| *PLCG1* | A01622 | IFNα/β |
| *PML* | A00951 | IFNα/β, IFNγ |
| *PTAFR* | A00928 | IFNγ |
| *SERPING1* | A00357 | IFNα/β, IFNγ |
| *SP100* | A01004 | IFNα/β |
| *STAT1* | A01784 | IFNα/β, IFNγ |
| *STAT2* | A00458 | IFNα/β, IFNγ |
| *THBS1* | A00847 | IFNγ |
| *TNF* | A00266 | IFNγ |
| *TNFRSF6* | A01081 | IFNα/β |
| *VCAM1* | A00301 | IFNγ |
| *VEGF* | A02182 | IFNα/β |
| *VIM* | A02181 | IFNγ |
| *WARS* | A00201 | IFNα/β, IFNγ |
